# Molecular Genetics of Retrovirus Replication

**DOI:** 10.3390/v15071549

**Published:** 2023-07-14

**Authors:** Judith G. Levin, Karin Musier-Forsyth, Alan Rein

**Affiliations:** 1 Eunice Kennedy Shriver National Institute of Child Health and Human Development, National Institutes of Health, Bethesda, MD 20892, USA; 2 Department of Chemistry and Biochemistry, The Ohio State University, Columbus, OH 43210, USA; 3 Center for Retrovirus Research, The Ohio State University, Columbus, OH 43210, USA; 4 Center for RNA Biology, The Ohio State University, Columbus, OH 43210, USA; 5 HIV Dynamics and Replication Program, National Cancer Institute-Frederick, National Institutes of Health, Frederick, MD 21702, USA

Despite the availability of effective anti-HIV drug therapy, according to UNAIDS estimates, 1.3 million people became newly infected with HIV and 630,000 died from AIDS-related illnesses in the year 2022 [1]. Clearly, there is still an urgent need for new discoveries that could stimulate development of novel antiviral strategies. To provide molecular insights into the major issues that still confront the HIV-AIDS field, we have assembled a collection of reviews that focus on key aspects of the “Molecular Genetics of Retrovirus Replication” and highlight current trends in research on steps in the virus life cycle.

Retrovirus infection is initiated when the viral envelope protein (Env) binds to receptors on the surface of the host cell; this interaction leads to the fusion of the viral membrane with the plasma membrane, so that the contents of the virion are deposited in the cytoplasm of the cell. The contents include the cone-shaped particle known as the viral “capsid” or “core”. The capsid is composed of ~2000 copies of the structural protein known as capsid (CA); it also contains the viral genomic RNA, which is coated and condensed by the HIV-1 nucleocapsid protein (NC), and the Pol proteins, reverse transcriptase (RT) and integrase (IN). RT catalyzes synthesis of a linear double-stranded (ds) DNA copy of the viral RNA (vRNA) genome in a reaction occurring within the core. Ultimately, the capsid must disassemble, releasing the newly synthesized DNA, which is then integrated into host DNA by IN [2,3,4,5,6,7,8,9,10].

In the present collection, Shen et al. [11] and Ingram et al. [12] describe exciting developments in our understanding of the disassembly process, including new evidence that (i) the intact HIV-1 core enters the nucleus by traversing the nuclear pore; (ii) synthesis of dsDNA is completed within the nucleus near the site of integration following nuclear entry; (iii) once disassembly of the capsid is initiated, the process is completed very rapidly; and (iv) viral DNA (vDNA) is released and is then integrated into host genomic DNA. Shen et al. [11] also detail the activities of the nuclear pore complexes, which they refer to as the “gatekeepers” at the nuclear envelope. These complexes contain about 30 nucleoporins (NUPs) such as NUP 358, which interacts with the capsid on the cytoplasmic side of the nuclear pore, and NUP 153, located on the nuclear side. In addition, Ingram et al. [12] point to the importance of microtubular proteins such as tubulin and dynein as well as the kinesin motor protein KIF5BL, which facilitate the cytoplasmic movement of the viral capsid along the microtubules in its journey to the nuclear pore.

Important aspects of the integration reaction are covered in two reviews. Engelman and Kvaratskhelia [3] discuss the properties and 3D architecture of HIV IN and highlight recent work showing that IN binding to vRNA is essential for generating a proper cone-shaped viral core. They point out that of the three classes of IN mutants (class I, II, and III), class II mutants all form eccentric particles and are blocked in steps after integration. However, since the specific defects that lead to this phenotype can vary, the authors propose sub-dividing class II mutants into class IIa, IIb, and IIc. To achieve successful retroviral replication, it is also critical that integration be restricted to a specific form of the viral genome, namely, a linear dsDNA with a sequence known as the long terminal repeat (LTR) at the 5′ and 3′ ends of the DNA, respectively. Viral DNA that enters the nucleus is part of a pre-integration complex (known as a PIC). Both unintegrated linear and circular forms of vDNA (1- and 2-LTR circles) must be silenced. Goff [13] reports that host cell strategies for silencing unintegrated murine leukemia virus (MLV) and HIV-1 vDNA in the nucleus are similar, although not identical. In both cases, there is rapid loading of histones associated with low levels of histone acetylation as well as participation of a histone methyltransferase (SetDB1/ESET). For silencing MLV, however, additional proteins including the HUSH complex, which contains three subunits, and NP220, a dsDNA binding protein, are also required.

Integration is followed by viral mRNA synthesis, viral protein synthesis, and assembly of progeny virions. Krebs et al. [14] review virus assembly and point to the central role played by the Gag polyprotein, which is cleaved by the viral protease to yield the major retroviral structural proteins as the virus buds from the plasma membrane. The features of the immature Gag lattice are discussed and possible mechanisms to explain the radical structural changes that occur as the mature hexameric lattice is formed are also presented. Ricaña and Dick [15] summarize what is known about a small, essential co-factor, inositol hexakisphosphate (IP6), which plays a seminal role in assembly and reverse transcription of HIV-1 and other retroviruses. IP6 activity is based on its binding to a ring of arginines in CA, stabilizing the viral core. The authors also describe the cellular pathway for synthesis of IP6 as well as IP5, which can partially substitute for IP6. Sumner and Ono [16] focus on the HIV-1 matrix (MA) domain in Gag and its role in virus assembly. They point out that the MA N-terminal myristoylated glycine and the downstream group of basic amino acids (collectively known as the “highly basic region” or HBR) are critical for promoting Gag binding to a specific lipid component of the plasma membrane, phosphatidylinositol (4,5) biphosphate (PI(4,5)P_2_) and also discuss how tRNA binding to the MA HBR regulates the specificity of this interaction. Trafficking of the HIV-1 Env protein through the cytoplasm to the plasma membrane and host factors that participate in this complex process are described in great detail in the review by Anokhin and Spearman [17]. Not all the steps are fully understood. For example, it is remarkable that when the Env precursor protein, gp160, reaches the plasma membrane, it does not interact with Gag to assemble a virus particle; instead, it undergoes endocytosis and re-sorting in the cytoplasm and only then, is incorporated into immature virions at the site of assembly.

Finally, our collection includes a comprehensive review by Hogan and Johnson [18] on the biological properties of gammaretrovirus-type envelope proteins broadly present in exogenous and endogenous retroviruses of many species, in other groups of viruses (e.g., Ebola, a filovirus), and even adapted for cell function (e.g., the placental syncytins, human SYNC1 and SYNC2).

In summary, in this Special Issue, we present reviews on major aspects of retrovirus replication, which we hope will stimulate further advances in the field. We thank all the authors for their thoughtful and informative contributions that made this Special Issue on “Molecular Genetics of Retrovirus Replication” possible. 
